# Hypoxia-Induced Oxidative Stress Modulation with Physical Activity

**DOI:** 10.3389/fphys.2017.00084

**Published:** 2017-02-13

**Authors:** Tadej Debevec, Grégoire P. Millet, Vincent Pialoux

**Affiliations:** ^1^Department of Automation, Biocybernetics and Robotics, Jozef Stefan InstituteLjubljana, Slovenia; ^2^Faculty of Biology and Medicine, Institute of Sport Sciences, University of LausanneLausanne, Switzerland; ^3^Laboratoire Interuniversitaire de Biologie de la Motricité EA 7424, Univ Lyon, Université Claude Bernard Lyon 1Villeurbanne, France; ^4^Institut Universitaire de FranceParis, France

**Keywords:** redox balance, altitude, hypoxemia, exercise, prooxidant, antioxidant

## Abstract

Increased oxidative stress, defined as an imbalance between prooxidants and antioxidants, resulting in molecular damage and disruption of redox signaling, is associated with numerous pathophysiological processes and known to exacerbate chronic diseases. Prolonged systemic hypoxia, induced either by exposure to terrestrial altitude or a reduction in ambient O_2_ availability is known to elicit oxidative stress and thereby alter redox balance in healthy humans. The redox balance modulation is also highly dependent on the level of physical activity. For example, both high-intensity exercise and inactivity, representing the two ends of the physical activity spectrum, are known to promote oxidative stress. Numerous to-date studies indicate that hypoxia and exercise can exert additive influence upon redox balance alterations. However, recent evidence suggests that moderate physical activity can attenuate altitude/hypoxia-induced oxidative stress during long-term hypoxic exposure. The purpose of this review is to summarize recent findings on hypoxia-related oxidative stress modulation by different activity levels during prolonged hypoxic exposures and examine the potential mechanisms underlying the observed redox balance changes. The paper also explores the applicability of moderate activity as a strategy for attenuating hypoxia-related oxidative stress. Moreover, the potential of such moderate intensity activities used to counteract inactivity-related oxidative stress, often encountered in pathological, elderly and obese populations is also discussed. Finally, future research directions for investigating interactive effects of altitude/hypoxia and exercise on oxidative stress are proposed.

## Introduction

Reactive oxygen species (ROS) and reactive nitrogen species (RNS) are constantly produced within the living cells (Allen and Tresini, 2000). The imbalance between antioxidants and prooxidants in favor of the latter, which results in disruption of redox signaling and/or molecular damage as a consequence of ROS and RNS overproduction, is referred to as oxidative stress (Sies and Jones, 2007). In order to counteract oxidative stress and thereby maintain the prooxidant/antioxidant balance, humans possess an elaborate antioxidant defense system comprising of endogenous antioxidant enzymes and non-enzymatic antioxidants (Powers and Jackson, 2008). Increased oxidative stress levels have been associated with numerous pathophysiological mechanisms (Zuo et al., 2015). In particular, excessive ROS production has been implicated in the development and exacerbation of pulmonary (Zuo et al., 2012), cardiovascular (Dhalla et al., 2000), metabolic (Roberts and Sindhu, 2009), and neurodegenerative (Sorce and Krause, 2009) disorders, as well as cancer (Liao et al., 2007).

It is of note however, that although excessive ROS production can lead to harmful effects including DNA damage and lipid peroxidation (Zuo et al., 2015), ROS and NOS are important agents involved in cell signaling (Sen and Packer, 1996) and mediate cell growth, repair and gene expression (Valko et al., 2007). ROS are also involved in cellular stress responses (Allen and Tresini, 2000) and in the systemic defense responses to pathogens (Tschopp, 2011). In addition, ROS production has been shown to play an important role in physiological adaptations to exercise. Numerous studies demonstrated that attenuating exercise-induced oxidative stress via antioxidant supplementation during training interventions blunted beneficial training-related adaptations of the skeletal (Ristow et al., 2009) and cardiovascular (Gliemann et al., 2013) system. Moreover, elevated ROS levels are also involved in stabilization of hypoxia-inducible factor-1α (HIF-1α) (Guzy et al., 2005), which is crucial for its function as the chief regulator of cellular responses to low O_2_ availability. Finally, the importance of ROS as messenger and signaling molecules, involved in cellular muscle adaptations to both, exercise (Allen and Tresini, 2000) and muscle disuse (Ji et al., 2006), is also well established. Based on the above it is clear, that the changes in oxidative stress and redox balance induce important, long-reaching effects on human health and performance.

Redox balance is importantly modulated by external factors such as environmental hypoxia (Magalhães et al., 2005) and physical activity (Ji, 1996). Both of these have been shown to augment oxidative stress in a dose-dependent manner (Goto et al., 2003; Debevec et al., 2015). On the other hand, hypoxia and exercise are also involved in improvement of antioxidant capacity and might therefore beneficially influence redox balance (Powers et al., 2016). While for long, the interactive effects of hypoxia and exercise on oxidative stress modulation did not received much attention, a number of recent studies scrutinized the potential combined effects of both factors (Quindry et al., 2016). Given the importance of redox balance for performance and health, these interactive effects are not only of interest for athletes employing different hypoxic training modalities but are also highly pertinent for certain patient population (e.g., heart failure, chronic obstructive pulmonary disease) that are concomitantly hypoxic and inactive.

The purpose of this review is to briefly summarize recent findings on hypoxia-related oxidative stress modulation by different activity levels. In addition, the paper also explores the applicability of moderate activity as means of attenuating hypoxia-induced oxidative stress and the potential of moderate activity to counteract inactivity-related oxidative stress, often encountered in pathological, elderly and obese populations.

## Altitude/hypoxia-induced oxidative stress

Hypoxia associated with high altitude exposure is known to elicit excess ROS and RNS production and thereby alter redox balance (Magalhães et al., 2005). Indeed, both acute (Magalhães et al., 2004; Pialoux et al., 2009d; Faiss et al., 2013) as well as long-term hypoxic exposures (Joanny et al., 2001; Askew, 2002; Dosek et al., 2007) have been shown to augment oxidative stress. Although increased oxidative stress has been observed both in response to hypobaric (i.e., terrestrial altitude) (Magalhães et al., 2005) as well as normobaric hypoxia (i.e., simulated altitude) (Debevec et al., 2014) recent evidence suggest that hypobaric hypoxia might induce higher oxidative stress levels than normobaric hypoxia (Faiss et al., 2013; Debevec et al., 2015; Ribon et al., 2016). While several differences in physiological responses between normobaric and hypobaric hypoxia have previously been reported (Millet et al., 2012) three distinct mechanisms seem directly involved in ROS modulation. First, ventilation is lower in hypobaric than normobaric hypoxia, with a lower tidal volume and a higher respiratory frequency (Savourey et al., 2003; Faiss et al., 2013). The higher alveolar physiological dead space in hypobaric hypoxia is also associated with ventilatory alkalosis and hypocapnia. Second, greater hypoxemia induced by hypobaric hypoxia could also play a role as negative correlation between hemoglobin oxygen saturation and oxidative stress levels has been demonstrated (Bailey et al., 2001). Finally, lower levels of exhaled NO were reported in hypobaric as compared to normobaric hypoxia (Hemmingsson and Linnarsson, 2009). This might be underlined by a higher NO back-diffusion to the alveoli and subsequently to the hemoglobin in hypobaric compared to normobaric hypoxia, suggesting that hypobaria promotes higher levels of NO recapturing by the blood compartment.

Oxidative stress response to environmental hypoxia depends on both the intensity and the duration (i.e., hypoxic dose) (Debevec et al., 2014, 2015). In fact, it seems that significant deleterious effects of hypoxia-induced oxidative stress are limited to high hypoxic doses (i.e., prolonged exposure to high altitude). It is also important to note that exogenous antioxidant supplementation does not seems to counteract hypoxia-induced oxidative stress during extended high altitude exposures (Subudhi et al., 2004). Furthermore, given the established role of oxidative stress in hypoxic ventilatory response modulation (Pialoux et al., 2009a), the use of antioxidant supplementation might even prove harmful and blunt ventilatory acclimatization to hypoxic condition. While the underlying mechanisms of hypoxia-induced ROS overproduction are not entirely clear, reductive stress within the mitochondria (Duranteau et al., 1998), augmented catecholamine production (Mazzeo et al., 1998), decreased mitochondria redox potential (Kehrer and Lund, 1994) and xanthine oxidase pathway activation (Yuan et al., 2004) are likely involved in this phenomenon.

## Physical activity and oxidative stress

The fact that acute exercise induces oxidative cell damage and thereby contributes to systemic oxidative stress has been initially established almost four decades ago by Dillard et al. (1978). Up-to-date investigations have clearly shown that both, acute and chronic exercise training increases oxidative stress levels predominantly within the skeletal muscle and the blood (Powers et al., 2011a, 2016). However, it is important to note that exercise modulates oxidative stress in a dose-dependent manner (Goto et al., 2003). In particular, the oxidative stress response magnitude does seem to be mainly related to the relative exercise intensity (i.e., higher intensity, higher exercise-related oxidative stress) and to a lesser extent the exercise duration (Johnson et al., 2012). Also, the magnitude of redox balance alterations differs between various exercise modes (e.g., endurance, resistance or concurrent) although they all seem to promote both, ROS overproduction and antioxidant system up-regulation (Azizbeigi et al., 2014). Several mechanisms have been proposed to explain the causes of exercise-induced ROS overproduction (Powers and Jackson, 2008). In particular, significantly increased O_2_ delivery to the working muscles can increase superoxide anion (${\text{O}}_{2}^{◦-}$) generation and other oxygen-derived intermediates (Sen, 1995) production in the mitochondria, and thereby promote oxidative damage within the cells (Guzy and Schumacker, 2006). Furthermore, exercise-induced increase in oxidative phosphorylation and catecholamine release are also potent sources of free radicals in response to physical exercise (Urso and Clarkson, 2003). Significantly increased phagocytic white cells concentration in the blood, following (eccentric) muscle damaging exercise is another source of exercise-related ROS overproduction (McArdle et al., 2004). In addition, ischemic reperfusion activates the xanthine oxidase within the endothelium and anti-inflammatory-related changes in NADPH oxidase have also been shown to generate large amounts of ${\text{O}}_{2}^{◦-}$ during physical exercise (Gomes et al., 2012). Finally, while exercise-induced oxidative stress within the tissues seems to be adequately reflected in the blood (Margaritelis et al., 2015), its role as an reactive species generator and redox balance modulator during exercise needs to be taken into account (Nikolaidis and Jamurtas, 2009).

While acute exercise of sufficient intensity is known to elicit increased oxidative stress, chronic exercise training seems beneficial for restoring redox balance (Radak et al., 2008). Chronic exercise was shown to significantly up-regulate primary antioxidant enzymes concentration within the skeletal and cardiac muscles (Powers et al., 2016). This exercise-related increase in antioxidant capacity also seems dose-dependent (Criswell et al., 1993) and exerts an important cardio-protective effect (French et al., 2008). It is therefore not surprising that highly trained endurance athletes have higher enzymatic antioxidant defense than their less trained counterparts (Marzatico et al., 1997). However, regardless of their higher baseline antioxidant capacity, the antioxidant system can also be importantly impaired in highly trained individuals following acute and chronic high-intensity or overload exercise training (Palazzetti et al., 2003).

Inactivity or muscle unloading represent the other side of the physical activity spectrum. However, similarly to exercise, inactivity seems to promote free radical, ROS and RNS overproduction and can also blunt antioxidant capacity (Laufs et al., 2005; Powers et al., 2012). It has been demonstrated that both, whole body (Dalla Libera et al., 2009; Agostini et al., 2010; Rai et al., 2011) and regional/limb unloading (Reich et al., 2010) result in augmented oxidative stress and altered redox balance. While the mechanisms of inactivity-induced oxidative stress seem multifactorial and are currently not fully understood (Powers et al., 2011b), alterations in muscle protein synthesis/proteolysis are likely to be among the key modulators (Powers et al., 2007). It is also important to note that increased systemic and local (muscular) levels of oxidative stress can significantly blunt muscle protein re-synthesis rate (Zhang et al., 2009) and promote proteolysis within the skeletal muscles (Smuder et al., 2010), which in turn result in muscle atrophy (Powers et al., 2011b). This is especially important in regards to the aging populations where inactivity-induced oxidative stress might be one of the central drivers of age-related sarcopenia (Derbre et al., 2014).

## Interactive effects of hypoxia and exercise on oxidative stress

As mentioned previously, both exercise (Ji, 1996) and hypoxia (Magalhães et al., 2005) can acutely augment oxidative stress. Recently, investigations also focused on the potential interactions between these two stressors (Quindry et al., 2016). It is nowadays well established that similarly to exercise performed in normoxia, hypoxic exercise induces ROS and NOS overproduction and increases markers of oxidative stress (Powers and Jackson, 2008). Importantly, acute hypoxic exercise of high-intensity (Møller et al., 2001; Pialoux et al., 2006) as well as moderate/low-intensity (Vasankari et al., 1997) does seem to augment oxidative stress. When interpreting the intensity-related aspects of hypoxic exercise, one also has to keep in mind that for the same absolute intensity the relative workload significantly increases as a function of reduced O_2_ availability in hypobaric or normobaric hypoxic conditions. Collectively, the data from the above studies suggest that at least at altitudes up to 5,000 m (or corresponding simulated altitudes), exercise likely drives more oxidative stress than systemic hypoxia *per se*. This hypothesis is congruent with the findings of Sinha et al. (2009) showing that graded exercise to exhaustion exerts a significantly greater additive increase in multitude of oxidative stress markers as compared to altitude exposure only.

Besides increasing oxidative stress, acute hypoxic exercise may also, at least transiently, alter antioxidant capacity (Quindry et al., 2016). In particular, studies indicate that an acute bout of hypoxic exercise augments circulating levels of uric acid (Sinha et al., 2009; Peters et al., 2016) known to be one of the key antioxidants involved in ROS and RNS plasma scavenging capacity (Cao and Prior, 1998). The uric acid increase following strenuous hypoxic exercise has also been shown to be related to an increase in plasma ferric reducing antioxidant potential (FRAP) (Quindry et al., 2008). Taken together, and in line with recent comprehensive review on the topic (Quindry et al., 2016), acute hypoxic exercise might concomitantly promote ROS and RNS production and increase antioxidant capacity. The latter mechanism is especially prominent when exercise bout is performed at low absolute exercise intensities [i.e., ≤ 60% peak oxygen consumption (VO_2peak_)]. The following section recaps the up-to-date studies scrutinizing the influence of activity during prolonged hypoxic/altitude exposures.

Several studies assessed the redox balance changes in response to chronic exercise training during prolonged high altitude sojourns (Miller et al., 2013; Lewis et al., 2014) or within the context of endurance events held at high altitudes (Quindry et al., 2008). However, given the numerous potential confounding factors inherent to field investigations, the present review was limited to the studies performed in a controlled and standardized manner. In particular, the Medline and Web of Science databases were utilized for the identification of pertinent papers using the following keywords: hypoxia or altitude, exercise or training, combined with oxidative stress and/or antioxidant capacity. The search yielded six eligible studies that also reported activity and nutritional-intake levels. These studies are summarized in Table 1. One of the first studies that investigated the effects of prolonged hypoxic exposure and concomitant daily exercise was performed at Pikes Peak high altitude research facility (Subudhi et al., 2004). While their purpose was to assess the effectiveness of antioxidant cocktail supplementation comprising β-carotene, α-tocopherol acetate and ascorbic acid on reducing altitude-induced oxidative stress, the study clearly indicated that high altitude residence augments blood and urine markers of oxidative stress. In addition, this study reported that prolonged submaximal exercise (2 h @ 50% VO_2peak_) performed upon acute exposure and following 13-day of acclimation did not increase oxidative stress. It is also important to note that the employed oral antioxidant supplementation was inefficient in reducing hypoxia-induced oxidative stress. Very informative data on the effects of chronic exercise training during prolonged hypoxic exposures on oxidative stress and antioxidant status have also been derived from investigations related to altitude training in (mostly) endurance athletes. The vast majority of these studies were performed using the Live-High Train-Low altitude training modality (LHTL) first introduced by Levine and Stray-Gundersen (1997), combining prolonged hypoxic exposures with concomitant chronic exercise training performed at lower altitudes. The initial study by Pialoux et al. (2009c) clearly showed that performing high intensity training sessions during 18-days exposure to simulated altitude significantly augments oxidative stress mostly via reduced antioxidant capacity (Table 1). A follow-up study indicated that the blunted antioxidant capacity might persist up to 2 weeks following the training camp (Pialoux et al., 2010). The fact that repetition of high-intensity exercises during LHTL training increases oxidative stress was also confirmed in a later study investigating potential differences between the normobaric and hypobaric LHTL protocols in highly trained triathletes (Debevec et al., 2015). While both protocols elicited significant disturbances in redox balance, it is of note that the LHTL protocol performed in hypobaric (terrestrial) hypoxia induced higher oxidative stress levels, most probably due to a higher overall hypoxic dose.

**Table 1:** **Summary of the key findings from the controlled studies on the combined effects of prolonged hypoxia and physical activity on oxidative stress and antioxidant markers**.

**Study setup/participants**	**Exercise**	**Oxidative stress markers**	**Antioxidant markers**	**Conclusions**	**References**
13-day HH exposure to 4,300 m	Daily habitual activity—non-structured	↑ LPO ↓ 8-OhdG	↑α-tocopherol ↑β-carotene	Hypoxia augments oxidative stress.	Subudhi et al., 2004
Healthy untrained individuals (*N* = 18)	2 × 2-h cycling @ 55% VO_2peak_ Days 1 and 13			No additive effect of exercise.	
18-day LHTL in NH (2,500–3,000 m) Control group LLTL (1,100 m) Elite runners (*N* = 12)	1-h·day^−1^—high intensity running (70–90% VO_2peak_)	Only Control (Following exercise test) ↓ MDA ↓ AOPP	Both groups ↓ FRAP ↓α-tocopherol ↓β-carotene ↓ lycopene	LHTL associated with high intensity training significantly augments oxidative stress.	Pialoux et al., 2009c
13-day LHTL in NH (2,500–3,000 m) Control group LL-TL (1,100 m) Elite swimmers (*N* = 18)	4-h·day^−1^—low-moderate intensity swimming (50–70% VO_2peak_)	Both groups (Following exercise test) ↓ MDA ↓ AOPP No differences at rest	Both groups ↔ FRAP ↔α-tocopherol	LHTL associated with moderate intensity training does not alter redox balance.	Pialoux et al., 2009b
18-day LHTL in HH (2,225 m) or 18-day LHTL in NH (≈2,225 m)—Well-trained trathlethes (*N* = 24)	3-h·day^−1^—moderate-high intensity running, cycling, swimming (70–90% VO_2peak_)	HH group ↓ AOPP ↑ Nitrotyrosine	HH group ↑ SOD ↑ UA NH group ↓ FRAP ↑ Catalase	LHTL associated with high intensity training significantly augments oxidative stress. HH LHTL seems to provoke higher oxidative stress than NH LHTL.	Debevec et al., 2015
10-day NH exposure to ≈4,000 m Healthy untrained individuals Training gr. (*N* = 8) Control gr. (*N* = 6)	2 × 1 h·day^−1^ moderate intensity cycling (≈50% VO_2peak_)	Control group ↑ AOPP ↑ Nitrotyrosine	Exercise group ↑ SOD ↑ Catalase ↑ GPX Control group ↑ GPX	Two hours of moderate intensity exercise per day blunts prolonged hypoxia-induced oxidative stress.	Debevec et al., 2014
10-day NH exposure to ≈4,000 m Healthy untrained females NBR group (*N* = 11) HBR group (*N* = 12) HAMB gr. (*N* = 8)	NBR and HBR—bed rest-induced inactivity. HAMB—2 × 20 min·day^−1^ low intenisty stepping, cycling (≈20–40% VO_2peak_)	NBR ↑ AOPP ↑ MDA HBR ↑ AOPP HAMB ↓ Nitrotyrosine	HBR ↑ Catalase ↓ GPX HAMB ↑ SOD ↑ Catalase	Hypoxia additively augments oxidative stress during inactivity. Habitual activity levels seem to blunt hypoxia-induced oxidative stress.	Debevec et al., 2016

*HH, Hypobaric hypoxia; NH, Normobaric hypoxia; VO_2peak_, peak oxygen consumption; LPO, Lipid hydroperoxides; 8-OHdG, 8-hydroxydeoxyguanosine; MDAm, malondialdehyde; AOPP, advanced oxidation protein products; FRAP, ferric-reducing antioxidant power; SOD, superoxide dismutase; UA, Uric acid; GPX, glutathione peroxidase; LHTL, Live-High Train-Low; LLTL, Live-Low Train-Low; NBR, Normoxic bed rest; HBR, Hypoxic bed rest; HAMB, Hypoxic ambulatory confinement; ↓, significantly decreased; ↑, significantly increased; ↔, no significant changes*.

In contrast to the above, 13-day LHTL protocol in swimmers, using moderate/low intensity chronic exercise training did not affect antioxidant status or significantly alter redox balance (Pialoux et al., 2009b). This suggests that low intensity endurance training performed during the LHTL might act as an antioxidant system up-regulation stimulus and can thereby reduce hypoxia-induced oxidative stress. This is in line with acute investigations suggesting increased levels of certain endogenous antioxidants following both high (Sinha et al., 2009) and moderate intensity hypoxic exercises (Peters et al., 2016). This has been further supported by laboratory-based and strictly controlled hypoxic confinement studies scrutinizing the independent and additive effects of hypoxia and chronic exercise in healthy male (Debevec et al., 2014) and female (Debevec et al., 2016) individuals. It has to be noted however, that during the LHTL training camps (Pialoux et al., 2009b,c, 2010), the exercise sessions were performed in normoxic conditions. This is in contrast to prolonged confinement (Debevec et al., 2014, 2015, 2016) or terrestrial altitude studies (Subudhi et al., 2004) where the exercise sessions were performed in hypoxia. As mentioned above, this needs to be taken into account when interpreting the influence of the relative exercise intensity on redox balance responses.

Similarly to studies in trained athletes (Pialoux et al., 2009b), the moderate exercise intensity (2 h·day^−1^ @ 50% VO_2peak_) performed twice daily throughout the 10-day hypoxic confinement period was shown to improve antioxidant capacity and thereby blunt hypoxia-related oxidative stress in untrained individuals (Table 1). This was further corroborated by our recent investigation indicating that even a low intensity physical activity levels, simulating habitual daily activity as opposed to inactivity, can enhance endogenous antioxidant status in healthy females (Debevec et al., 2016). Taken together, these data suggest that during hypoxic exposures, physical activity significantly modulates systemic redox balance in humans. Whereas high intensity exercise performed during prolonged hypoxia leads to higher cumulative oxidative stress levels, moderate-low intensity physical activity may blunt hypoxia-induced oxidative stress. One of the key underlying mechanisms seems to be the exercise-induced up-regulation of enzymatic and non-enzymatic antioxidant capacity, especially augmented uric acid and FRAP reported following both acute (Sinha et al., 2009; Peters et al., 2016) and chronic (Debevec et al., 2014, 2016) hypoxic exposures.

## Inactivity, hypoxia and oxidative stress

As mentioned before, inactivity significantly affects redox balance (Laufs et al., 2005; Powers et al., 2012). While the combination of inactivity and hypoxia is rarely encountered in healthy individuals, both factors often characterize numerous chronic diseases and are also observed in aging population (Levine et al., 1997). Muscle unloading due to inactivity is often associated with chronic respiratory disease (Rittweger et al., 2016) and heart failure (Tsutsui et al., 2011), both known to also induce persistent systemic hypoxemia. It is well established that such chronic conditions increase ROS and RNS production as well as decrease antioxidant system capacity (White et al., 2006). Given the well-established detrimental effects of continuously elevated oxidative stress levels in many pathologic conditions, mitigation strategies aiming at reestablishing the redox balance are warranted (Zuo et al., 2015). Indeed, physical exercise may be one of the key stimuli in restoring prooxidant/antioxidant balance in chronic disease patients (Mercken et al., 2005). In this context, regular physical activity is likely superior to oral antioxidant supplementation in regards to redox balance restoration (Urso and Clarkson, 2003). The effectiveness of exercise is not surprising, given that inactivity-induced oxidative stress seems to be chiefly related to alterations in muscle protein synthesis/proteolysis (Powers et al., 2007).

Obesity is also often associated with combined inactivity and hypoxia. Besides having high relative levels of fat tissue, obese individuals also tend to be significantly less active as compared to their non-obese counterparts (Janssen et al., 2005). The cytokine release from the excessive white adipose tissue and various other processes related to metabolic syndrome are known to elicit continuously elevated levels of oxidative stress (Trayhurn et al., 2008). In addition, higher occurrence of obstructive sleep apneas in obese individuals (Bradley and Floras, 2009) is associated with significant local and systemic hypoxia episodes which again augment oxidative stress. Indeed, extensive body of literature, reviewed by Vincent and Taylor (2006) highlights significant correlation between obesity and oxidative stress. While oxidative stress reduction strategies in obese individuals include and combine activity, dietary, pharmacological and surgical interventions, exercise *per se* has been shown to be a potent modulator of beneficial redox balance adaptation (Vincent et al., 2007). Besides its effect on fat mass reduction, exercise also seems to reduce systemic oxidative stress by augmenting antioxidant system and improving glycemic control. Interestingly, exposure to environmental hypoxia (Kayser and Verges, 2013) as well as exercising in hypoxia (Millet et al., 2016) have recently been proposed as compelling treatment strategies for obesity and related metabolic abnormalities. However, careful application of hypoxia in obese and other patient populations, known to exhibit systemically elevated oxidative stress values, is warranted in order to avoid potential harmful environmental hypoxia-induced effects on redox balance.

Our recent study on the combined effects of inactivity and hypoxia in healthy females (Debevec et al., 2016) also provided some further insight into this topic. Briefly, it was shown that the addition of hypoxia (simulated altitude 4,000 m) during 10-day of bed rest-induced inactivity additively increases oxidative stress mostly via antioxidant capacity reduction. Contrastingly, as already mentioned in the previous section, hypoxia-induced oxidative stress was overrun by beneficial effects of habitual levels of physical activity. In summary, moderate to low intensity exercise training is likely beneficial for reducing elevated levels of oxidative stress caused independently or combined by inactivity and hypoxia. This is an important consideration in regards to numerous patient populations as well as aging individuals that are often inactive due to their underlying medical condition. Given that even low levels of physical activity might contribute to restoring redox balance, regular exercise should be encouraged in these populations.

## Perspectives

It is clear from the above-summarized studies that altered physical activity level differentially affects systemic oxidative stress and antioxidant capacity levels. There is strong experimental evidence, derived from well-controlled prolonged hypoxic studies, that low-to-moderate intensity exercise training exerts a positive influence on redox balance (i.e., reduced oxidative stress levels), mostly underlined by augmented antioxidant capacity. On the other hand, performing exercise of higher-intensities seems to additively increase hypoxia-induced oxidative stress. This might compromise adaptations to hypoxic training in athletes and also prove detrimental for individuals who exhibit chronically elevated systemic oxidative stress levels. These observations need to be taken into account while providing expert-advice on altitude/hypoxic training, as well as guidelines for high altitude sojourns in vulnerable populations. Given that the exact mechanisms of the interactive effects of hypoxia and physical activity on redox balance are not entirely clear, future well-controlled investigations should scrutinize different dose-response effects of both in healthy as well as patient populations.

## Author contributions

TD, GM, and VP: Drafted the manuscript, revised the manuscript critically for important intellectual content, and approved the final version of the manuscript submitted.

## Funding

This study was supported by the Institut Universitaire de France.

### Conflict of interest statement

The authors declare that the research was conducted in the absence of any commercial or financial relationships that could be construed as a potential conflict of interest.
